# A Case of Partial Placenta Prævia, Complicated with Transverse Presentation and Rigidity of the Os Uteri

**Published:** 1856-06

**Authors:** M. J. Greene

**Affiliations:** Loachapoka, Ala.


### ARTICLE II.

*A Case of Partial Placenta Prævia, complicated with transverse presentation and rigidity of the Os Uteri.* By M. J. GREENE, M. D., Loachapoka, Ala.

About 11 o'clock, A. M., on the 14th inst., I was called to a colored woman in labor, belonging to Mr. T. M. B———. Upon my arrival, I found that labor had made but little progress, the uterine contractions being slight; and as yet, my patient had exhibited no symptom indicative of complication or approaching danger.

The labor pains began soon to increase, and an examination "per vaginane" revealed the os uteri high up in the pelvis, dilated to about the size of a sixpense, firm and unyielding.

About 2 o'clock, P. M., a second examination was instituted. The os tincæ had somewhat descended, but, notwithstanding the pains had now become quite severe, I was yet unable to satisfy myself with regard to the presentation. Learning from Mr. B—— that she had usually been bled in her previous labors, I inferred that they had generally been attended by rigidity of the os uteri. In view of this highly probable fact, as my patient was plethoric, and as a means of overcoming the persistent disinclination to dilatation of the soft parts, I determined, at once, to bleed, which I did to the extent of producing a decided impression on the circulation, but short of syncope.

A temporary suspension, succeeded by an increase of the uterine contraction, was the result of the bleeding; and along with the general relaxation which followed, to my surprise and grief, I found that uterine hemorrhage had immediately set in. An examination now revealed the startling fact, that I had before me a case of partial placenta prævia, with transverse presentation—the right foot and hand presenting.

The hemorrhage continued, increasing upon every return of the pain, and partially subsiding during the interval.

The strength of the patient, as also the uterine contractions, began sensibly to decline, and being convinced that a cautious, but prompt, delivery afforded the only means of saving the life of the mother, I immediately reqnested a consultation with my friend, and associate, Dr. W. W. Bloodworth, who was promptly on the spot.

At my request, Dr. B. proceeded to examine the patient, and distinctly felt the placenta attached on the left side of the os, and the foot on the right. The labor pains having almost entirely subsided, we administered ergot in decoction, and upon the first manifestation of its specific effect, cautiously began the delivery by pushing back the hand and bringing down the foot.

Some difficulty was experienced in obtaining command of the other foot: this, however, was eventually accomplished, and the delivery was completed in a few moments.

The child, of course, was "still-born:" its death having occurred when the relaxation of the os tincæ produced a separation of the placenta from its uterine attachment.

Our attention was now directed to the extremely exhausted and restless condition of the mother. Sixty drops of tinct.

opii. were given. Fearing that the contractions of the uterus might not be sufficiently prompt and forcible to arrest the hemorrhage, a decoction of ergot was administered; external pressure being occasionally applied over the region of the womb.

The delivery having been completed about 11½ o'clock, P. M., we remained with our patient during the remainder of the night. She continued restless, with cold extremities, feeble circulation, &c., until 4 o'clock, A. M., when she became quiet, and slept well until 6 o'clock; extremities still cool; pulse feeble, 110. Ordered frictions and synapisms to the extremities, with such other remedial measures as the case required, and left the patient in the hands of the nurse.

The only untoward symptom which has materially interfered with the comfort of my patient since her delivery, is the characteristic headache, which usually follows exhausting sanguineous discharges from the uterus.

This was so extremely distressing as to preclude the possibility of rest or sleep. The usual remedies having failed to afford relief, a blister was applied to the shaven scalp. This, with due attention to the bowels, and a mild alterative course of treatment, was followed by a marked improvement. She is now slowly, but encouragingly convalescing.

*Remarks.*

Perhaps simple justice to myself demands a brief allusion to the item of venesection in the treatment of the above case. I readily admit that had I been forewarned of the condition of things which subsequently became developed, I might have omitted the use of the remedy. But it will be borne in mind, that up to the time of the bleeding, the rigidity had persisted to such an extent as utterly to preclude the possibility of introducing the finger into the os uteri.

The patient was plethoric; had habitually been bled previously; was in her fifth labor, and had never before suffered from uterine hemorrhage. So that the remedy is not only sanctioned by high obstetrical authority, but would, under precisely similar circumstances, have been unhesitatingly employed by any intelligent practitioner.

Indeed, it is a question worthy of consideration, whether the remedy was not in this case, at least, an innocent one, since a certain degree of relaxation of the soft parts was essential to the accomplishment of delivery, and the sooner the desired end could be attained the better for the patient. The quantity of blood drawn from the arm must, therefore, have materially hastened the period at which delivery was practicable and safe.

